# Regulation of *Caenorhabditis elegans *body size and male tail development by the novel gene *lon-8*

**DOI:** 10.1186/1471-213X-7-20

**Published:** 2007-03-20

**Authors:** Gwen Soete, Marco C Betist, Hendrik C Korswagen

**Affiliations:** 1 Hubrecht Laboratory, Netherlands Institute for Developmental Biology and Center for Biomedical Genetics, Uppsalalaan 8, 3584 CT, Utrecht, The Netherlands

## Abstract

**Background:**

In *C. elegans *and other nematode species, body size is determined by the composition of the extracellular cuticle as well as by the nuclear DNA content of the underlying hypodermis. Mutants that are defective in these processes can exhibit either a short or a long body size phenotype. Several mutations that give a long body size (Lon) phenotype have been characterized and found to be regulated by the DBL-1/TGF-β pathway, that controls post-embryonic growth and male tail development.

**Results:**

Here we characterize a novel gene affecting body size. *lon-8 *encodes a secreted product of the hypodermis that is highly conserved in Rhabditid nematodes. *lon-8 *regulates larval elongation as well as male tail development. In both processes, *lon-8 *appears to function independently of the Sma/Mab pathway. Rather, *lon-8 *genetically interacts with *dpy-11 *and *dpy-18*, which encode cuticle collagen modifying enzymes.

**Conclusion:**

The novel gene *lon-8 *encodes a secreted product of the hypodermis that controls body size and male ray morphology in *C. elegans*. *lon-8 *genetically interacts with enzymes that affect the composition of the cuticle.

## Background

The regulation of body size is an important yet poorly understood aspect of animal development [1]. Unlike vertebrate size, nematode body size is largely dependent on cell size rather than cell number [2-4]. Nevertheless, *Caenorhabditis elegans *is an attractive model to study the genetics of body size determination because many mutants that specifically affect size have been isolated and characterized. In *C. elegans*, the hypodermis, which is the epithelial structure that encapsulates the worm, is the primary site where growth is regulated. The hypodermis consists of several syncytia of fused epidermal cells [5]. The largest of these syncytia that covers the entire mid-body region of the animal is called hyp7. During larval development, hyp7 increases in nuclear content and cell size as progressively more daughters of the lateral seam cells fuse with it [4,5]. The syncytial nuclei undergo several rounds of endoreduplication during the fourth larval stage and adulthood [2,6]. In *C. elegans *and other nematode species, a substantial part of growth takes place after larval development is completed [2]. The amount of post-larval growth is correlated with the amount of endoreduplication that occurs in the hypodermis during adulthood [2,7]. Therefore, growth is at least partially dependent on the DNA content of the hypodermis [2,4,7].

Another factor that affects body size in *C. elegans *is the composition of the exoskeletal cuticle, which is synthesized by the hypodermis before hatching and at the end of each larval stage (reviewed in [8,9]). Mutations in cuticle components can result in dumpy (Dpy) or squat (Sqt) phenotypes, meaning animals are shorter and thicker than wild-type [10]. Many of these *dpy *and *sqt *genes encode collagens or the enzymes required to process them [8,9]. There are also mutations that affect body size without affecting morphology. In these cases the animals' size is altered but their proportions are more or less intact, resulting in small (Sma) or long (Lon) phenotypes [10]. So far, only two *lon *genes have been cloned and characterized. Both genes encode molecules that are synthesized and secreted by the hypodermis. *lon-3 *encodes a cuticle collagen that is required for body size regulation primarily during late larval development [11,12]. *lon-1 *encodes a member of the CRISP (Cysteine-Rich Secretory Protein) family of unknown function [13,14]. *lon-1 *animals are of wild-type length at hatching, but grow substantially faster than wild-type during both larval and adult stages. Part of the increase in body size of *lon-1 *animals is proposed to be due to an increase in the ploidy of the hypodermal nuclei [14].

In *C. elegans*, growth is controlled by a highly conserved TGF-β pathway, the Sma/Mab (for Small/Male abnormal) pathway (reviewed in [15]). Mutants of components of this pathway are smaller than wild-type, but not obviously dumpy. The ligand, DBL-1, is a dose dependent regulator of body size. *dbl-1 *loss-of-function causes a Sma phenotype, whereas overexpression causes a Lon phenotype [16,17]. Mutants of *dbl-1 *or positive downstream regulators of the Sma/Mab pathway are of wild-type length at hatching, but by the time they reach adulthood they are about a third shorter [16-20]. In addition, adult growth and endoreduplication of the hypodermal nuclei is reduced in *dbl-1 *animals [7]. Therefore, the Sma/Mab pathway is required for larval elongation as well as adult growth.

The effects of *dbl-1 *signaling are mediated in part through the regulation of *lon *genes. *dbl-1 *is thought to regulate translation, modification or degradation of the LON-3 protein [12], whereas *lon-1 *is a negatively regulated transcriptional target of DBL-1 [13,14]. In addition, mutations in *lon-3 *and *lon-1 *can suppress the small phenotype of Sma/Mab pathway mutants [11-14]. Here, we identify a novel regulator of body size, *lon-8*, which is also required for male tail morphology. *lon-8 *encodes a novel secreted protein that is highly conserved within the phylum *Nematoda*. Our results suggest that LON-8 regulates body size independently of the Sma/Mab pathway.

## Results

### *lon-8 *encodes a small protein that is likely to be secreted

**Figure 1:**
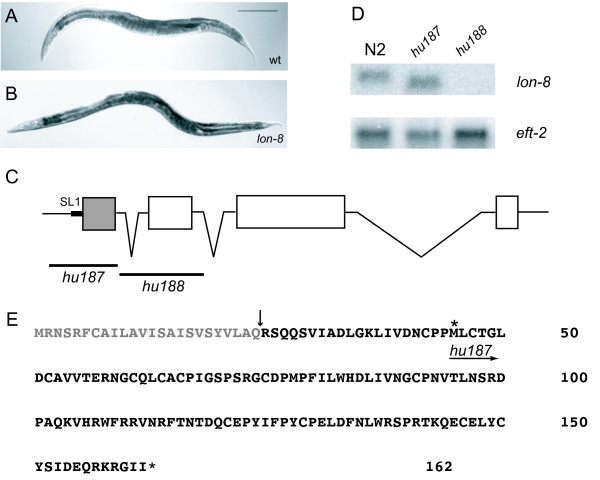
**Lon phenotype resulting from mutations in *lon-8***. (A) N2 wild-type hermaphrodite grown at 20°C for 120 hours. Bar is 200 μm. (B) *lon-8(hu187) *hermaphrodite grown at 20°C for 120 hours. (C) Structure of *lon-8 *gene. SL1 is the SL1 trans-splice leader sequence. The region which encodes the signal peptide is shaded. The *lon-8(hu187) *and *(hu188) *deletions are indicated. (D) Northern blot analysis; RNA was isolated from mixed-stage populations of animals. Blots were probed with the amplified full-length sequence of the *lon-8 *cDNA. *eft-2 *was used as a control for loading [13]. Animals containing *hu187 *express a transcript which is recognized by the *lon-8 *probe, but is shorter than the wild-type transcript. *hu188 *animals do not express any transcript recognized by the *lon-8 *probe. (E) LON-8 amino acid sequence. The signal sequence is indicated in grey and the predicted site of cleavage is shown. The asterisk indicates the initiator methionine of the predicted LON-8 truncation present in *hu187*.

We found that Y59A8B.20 RNAi by feeding causes an increase in body length (1.3 ± 0.08 mm in animals treated with control vector versus 1.4 ± 0.08 mm in animals treated with *lon-8 *RNAi, n = 75), as previously observed by others [21]. Upon amplification and sequencing of the Y59A8B.20 transcript from a *C. elegans *cDNA library, it became apparent that the sequence of this transcript differs from the predicted Y59A8B.20 gene structure in that it contains an additional final fourth exon (Figure 1). The complete Y59A8B.20 sequence (Genbank accession number EF495354) encodes a 162 amino acid protein containing an N-terminal signal sequence. There are no other conserved motifs such as transmembrane domains or signals for lipid mediated anchoring in the sequence. Y59A8B.20 is strongly expressed in the hypodermal syncytium (see below). Clear localization to this cell is only visible when the signal sequence is deleted, implying that in the presence of this sequence Y59A8B.20 is secreted and diffuses. Y59A8B.20 is therefore likely to be a secreted protein.

To investigate the loss-of-function phenotype further, we identified and isolated two deletions of Y59A8B.20 (see Methods). Both alleles gave a clear Lon phenotype, therefore Y59A8B.20 was named *lon-8*. The first allele generated, *hu187*, is a deletion of the entire *lon-8 *first exon and surrounding sequences. A shorter transcript can still be detected in *hu187 *animals by Northern blotting (Figure 1). We amplified this fragment by reverse transcription PCR (RT-PCR, see Methods) and sequenced it. The alternative transcript found in *hu187 *mutants is predicted to encode an N-terminal truncation of the full-length LON-8 protein starting from an in-frame methionine present in the second exon. This transcript cannot be detected in wild-type animals by Northern blotting or RT-PCR. Importantly, if the *hu187 *transcript were to be translated, the resulting protein would lack the signal peptide. Furthermore, treatment of *hu187 *mutants with *lon-8 *RNAi did not enhance the Lon phenotype (1.5 ± 0.08 mm in *hu187 *animals on control vector versus 1.4 ± 0.08 mm in *hu187 *animals treated with *lon-8 *RNAi, n = 75). Therefore, it seems unlikely that this shorter version of LON-8 is functional. The second deletion allele generated, *hu188*, lacks the entire second exon and surrounding sequences (Figure 1). Alternative splicing of *lon-8 *from the first to the third exon would result in a frame-shift leading to a nonsense transcript, moreover, no transcript can be detected in these animals by Northern blotting. Both alleles show similar phenotypes in the experiments described below. Taken together, these results strongly suggest that both alleles represent a strong loss-of-function or null phenotype.

### *lon-8 *loss-of-function affects body size during larval development and early adulthood

**Figure 2:**
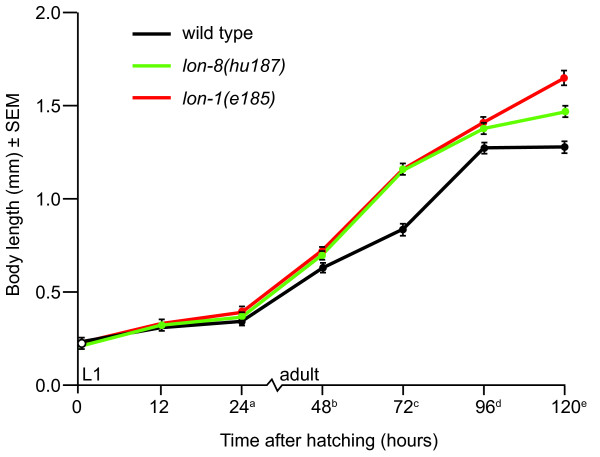
**Growth curve of *lon-8(hu187) *mutants**. Wild type, *lon-1(e185) *and *lon-8(hu187) *animals were synchronized and measured at seven time points. The animals are of indistinguishable length at hatching, but *lon-1 *and *lon-8 *animals grow faster than wild type animals throughout larval development and during early adulthood. Later in adult life, *lon-1(e185) *animals continue to grow at a faster rate (steeper slope angle) than wild type animals, whereas the growth rate of *lon-8(hu187) *animals stabilizes. At least 100 animals were measured for each strain. a, b, c, for wild type/*lon-1 *and wild type/*lon-8 *p < 0.001. d, e for wild type/*lon-1*, wild type/*lon-8 *and *lon-1*/*lon-8 *p < 0.001. P values were calculated using Student's t-test.

To determine at which developmental stages body length is affected in *lon-8 *mutants, we synchronized animals by collecting them an hour after hatching and letting them grow for different periods of time before measuring their length (Figure 2). We compared the *lon-8(hu187) *phenotype with that of *lon-1(e185) *animals, which grow more than wild-type animals during both larval development and adult growth [13,14]. At hatching, *lon-8(hu187) *mutants were no longer than wild-type animals, but throughout larval development, they outgrew their wild-type counterparts at a rate comparable to that of *lon-1(e185) *animals. At around 96 hours post-hatching, the growth rate of *lon-8(hu187) *slowed down compared to *lon-1 *animals, which kept growing at the same rate. This suggests that *lon-8 *is more important for body size determination during larval elongation and early adult growth than later in life.

As *lon-8 *acts at stages in development when there is cell proliferation in the hypodermis, it could be influencing the number of seam cells or the number of syncytial nuclei, which could lead to an increased body size [2,4,7]. We stained N2, *hu187 *and *hu188 *animals with DAPI and counted the number of hypodermal and seam cell nuclei located between the postdeirid sensillum and the anus to determine whether the number of hypodermal nuclei is elevated in *lon-8 *mutants compared to wild-type. We did not observe a difference in the number of hypodermal nuclei (in each case, 21 ± 1 nuclei, n = 20 for each strain). We also investigated whether the total number of seam cells is affected by crossing the seam cell specific GFP marker *wIs51 [scm::gfp] *[22] into the *hu187 *background and counted the total number of seam cells lining the flank of the worm. Again, we observed no difference in seam cell number between wild-type and *lon-8(hu187) *animals (16–17 seam cell nuclei, n = 32 animals for each strain). This suggests that the increase in body length of *lon-8 *animals correlates with an increase in cell size rather than cell number.

Adult growth in *C. elegans *is thought to be regulated by the number of endoreduplication rounds completed by the nuclei of the hypodermal syncytium [2,7]. In *lon-1 *mutants, some of the hypodermal nuclei undergo additional rounds of endoreduplication, leading to an increased ploidy compared to wild-type, and this is thought to contribute to their excessive growth [11,14]. In contrast, *lon-3 *mutants have no detectable increase in hypodermal ploidy [11]. To see if adult growth by hypodermal endoreduplication is affected in *lon-8 *mutants, we measured the DNA content of individual hypodermal nuclei in the posterior lateral hypodermis of synchronized populations of 120 hour old animals using DAPI staining and microdensitometry [2,23] as well as PI staining and confocal analysis [23]. We were unable to detect a difference in hypodermal ploidy between wild-type and *lon-8 *animals using either of these techniques (a ploidy of 7.9 ± 1.6 for N2 and 8.2 ± 2.4 for *lon-8(hu188) *when analyzing 10 animals using PI staining, see Methods for details). Taken together, these results lead us to conclude that *lon-8 *does not have an obvious effect on the nuclear content of the hypodermis.

### *lon-8 *is expressed in the hypodermis throughout development

**Figure 3:**
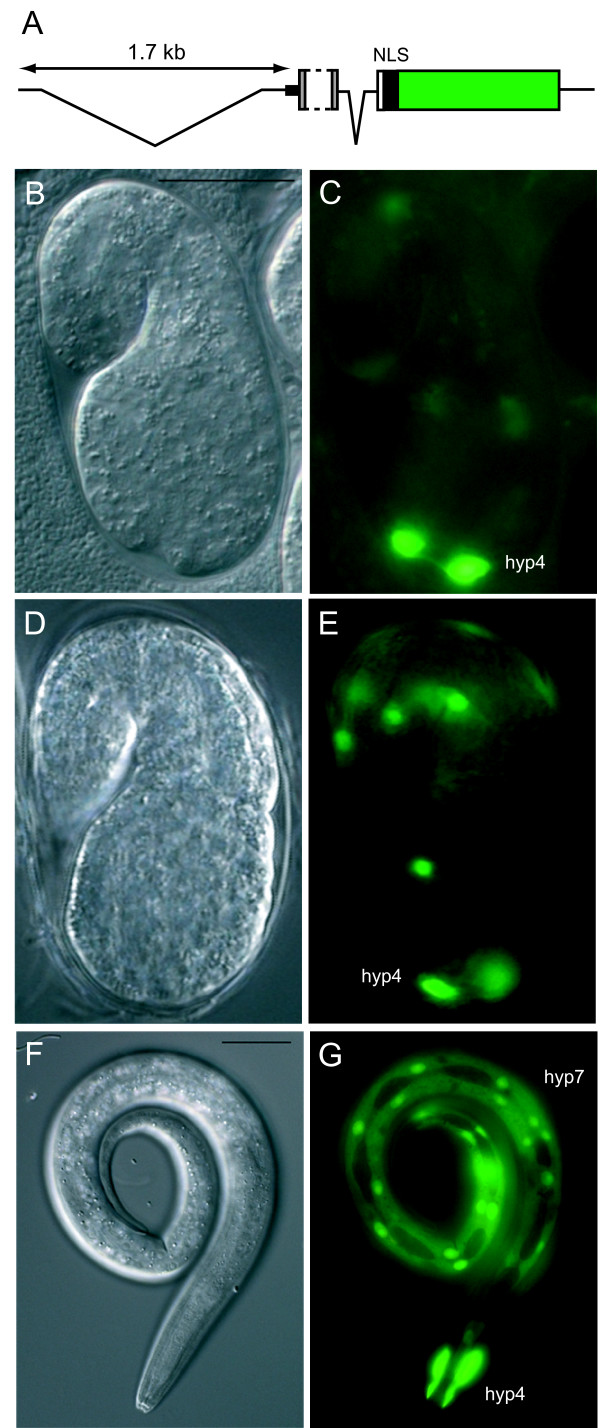
**Expression pattern of a *lon-8 *transcriptional reporter**. (A) Schematic representation of the reporter construct (see text for details). The portion of the first exon that is removed is indicated with a dashed line. The *gfp *coding sequence is shown in green. (B) to (G) Differential interference contrast (DIC) microscopy and corresponding fluorescence micrographs. Bar is 20 μm. (C) A comma stage embryo expressing *lon-8::gfp *in the nuclei of hyp4. (D), (E) A 1.5 fold stage embryo expressing *lon-8::gfp *in hyp4 and hyp7 nuclei. (F), (G) An L1 larva expressing *lon-8::gfp *in hyp4 and the hyp7 syncytium.

We also investigated where *lon-8 *function might be required for its role in body size determination. To this end, a transcriptional reporter was constructed, consisting of the *lon-8 *upstream sequence and part of the genomic sequence (Figure 3A; see Methods), which was inserted into a vector containing the coding sequence for nuclear localized GFP. Because the LON-8 N-terminus contains a signal peptide, which could cause the secretion and diffusion of GFP, another variant was made excluding the signal sequence. Multiple lines carrying extra-chromosomal arrays of these constructs were generated by microinjection. The lines carrying the variant of the reporter construct including the signal peptide mostly showed weak GFP expression in the posterior gut (data not shown) but nowhere else, presumably because the GFP is secreted and diffuses. However, the lines carrying the variant lacking the signal peptide showed clear expression in two of the hypodermal syncytia, hyp4, which surrounds the anterior part of the pharynx, and hyp7, which surrounds the mid-body region of the animal (Figure 3). The earliest detectable GFP expression can be observed in comma stage embryos, where there is abundant expression in the newly generated hyp4 nuclei and, albeit less frequently, in hyp7 nuclei. This expression pattern remains consistent throughout embryonic and larval development up until adulthood. There is faint expression in the hindgut, which most likely represents background expression of the reporter.

### *lon-8 *cannot suppress the Sma phenotype of *dbl-1 *mutants

**Figure 4:**
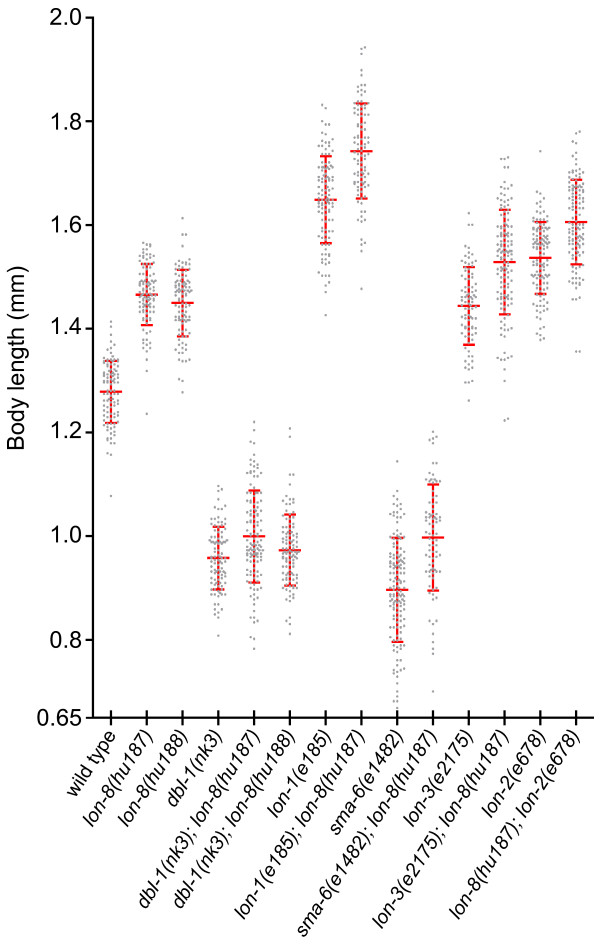
**Epistatic analysis of *lon-8 *and Sma/Mab mutants**. Animals of indicated genotypes were synchronized, grown at 20°C for 120 hours and measured as described in the Methods. At least 75 animals were measured for each strain. Each dot represents one animal. Average length and standard deviation are indicated.

Both *lon-1 *and *lon-3 *can suppress the small phenotype of Sma/Mab mutants [11,13,14]. Several *lon-1 *alleles even suppress the Sma phenotype so strongly that double mutant combinations can become longer than wild-type [13,14]. To determine whether *lon-8 *mutations can likewise suppress the phenotype of Sma mutants, we generated double mutant combinations of *hu187 *and *hu188 *with *dbl-1*, and of *hu187 *with *sma-6*, which encodes the Type I receptor for DBL-1 [18] (Figure 4). If *lon-8 *were an important downstream effector of *dbl-1*, one would expect that double mutants of *lon-8 *with *dbl-1(nk3) *or *sma-6(e1482) *would reach a body length longer than or equal to wild-type. Although *lon-8 *has an effect on the maximum length that *dbl-1 *animals can reach, there is no clear suppression of the Sma phenotype in *dbl-1(nk3) lon-8 *animals. In double mutants between *sma-6(e1482) *and *lon-8(hu187)*, there is a slightly stronger suppression of the Sma phenotype than in *dbl-1(nk3) lon-8(hu187) *animals, which could be due to the fact that *e1482 *is a hypomorphic allele of *sma-6*. In any case, both *dbl-1(nk3) lon-8(hu187) *and *sma-6(e1482); lon-8(hu187) *double mutants still show a strong and penetrant Sma phenotype. Taken together, these data indicate that *lon-8 *is unlikely to be an important effector of DBL-1 signaling. We also analyzed the body size phenotype of double mutant combinations of *lon-8 *and three other *lon *genes; *lon-2*, which functions upstream of *dbl-1 *in the Sma/Mab pathway [24], *lon-1 *and *lon-3*. In all cases, *lon-8(hu187) *enhanced the average single mutant body size to a similar degree, which is around 10%.

### *lon-8 *is not transcriptionally regulated by the Sma/Mab pathway

**Figure 5:**
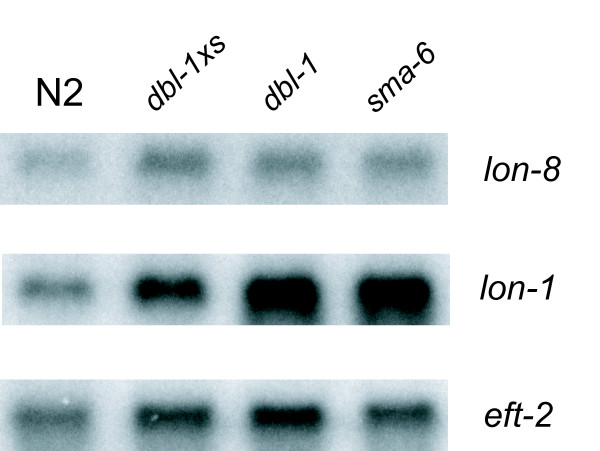
**Northern blot analysis of *lon-8 *and *lon-1 *mRNA levels in Sma/Mab mutant strains**. RNA was isolated from mixed populations of animals carrying *ctIs40 [dbl-1xs] *(which results in DBL-1 overexpression), *dbl-1(nk3) *or *sma-6(e1482)*. Blots were hybridized with probes corresponding to *lon-8*, *lon-1 *or the loading control *eft-2*. Whereas *lon-1 *mRNA levels are clearly increased in *dbl-1(nk3) *and *sma-6(e1482) *animals, we could not detect a difference in *lon-8 *mRNA levels by microdosimetry in four experiments with independently prepared RNA.

Two sets of experiments were carried out to further study the relationship between *lon-8 *and the Sma/Mab pathway. Firstly, we examined the expression of the *lon-8 *transcript in several *Sma/Mab *pathway mutants by Northern blotting (Figure 5). Others have shown that the *lon-1 *transcript is clearly upregulated in the Sma/Mab pathway mutants *dbl-1 *and *sma-6*, whereas it is downregulated in animals overexpressing *dbl-1*, thus establishing that *lon-1 *is a negatively regulated transcriptional target of *dbl-1 *signaling [13,14]. Whereas the *lon-1 *transcript is clearly upregulated in *dbl-1 *and *sma-6*, the expression level of the *lon-8 *transcript is similar to the wild-type level in these mutants. In addition, we crossed the extrachromosomal arrays of our transcriptional reporter construct without the signal peptide into *dbl-1(nk3)*. As some of our lines were generated using a red fluorescent injection marker expressed in the pharynx (see Methods), we could use these extrachromosomal arrays to compare the proportion of transgenic animals (with a red pharynx) that also expressed GFP in hyp4 and/or hyp7 in a wild type versus a *dbl-1 *background. We found no difference in the number of transgenic animals that expressed GFP under control of the *lon-8 *promoter between wild-type and *dbl-1 *animals (n > 100), nor could we detect any difference in GFP intensity by eye (data not shown). Taken together, these results imply that *lon-8 *is not transcriptionally regulated by the Sma/Mab pathway.

### *lon-8 *is required for male ray development

**Figure 6:**
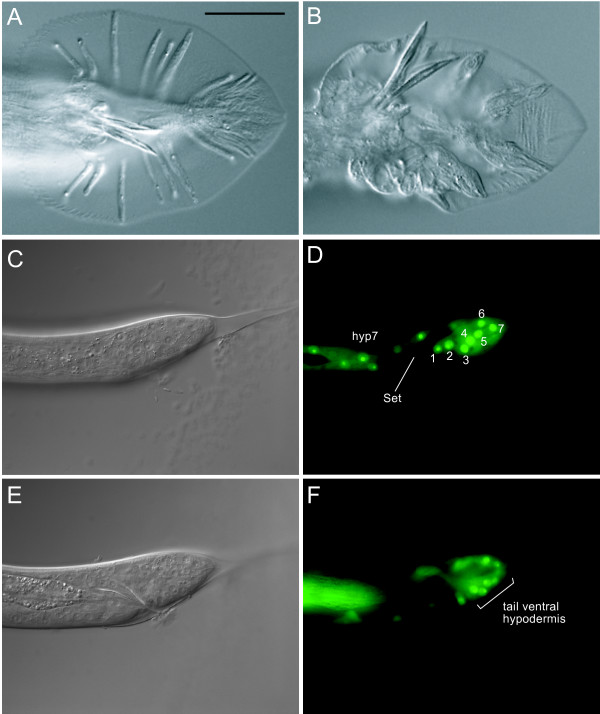
**Male tail phenotype resulting from mutations in *lon-8***. (A), (B), DIC micrographs of ventral views of representative male tails of *him-5(e1490) *and *lon-8(hu187) *animals. Bar is 20 μm. Whereas rays 1–9 can clearly be distinguished in wild type males, in *lon-8 *males they are clumped together as an amorphous mass. (C)-(F) DIC and fluorescence micrographs of approximately 40 hour old *him-5(e1490) *males expressing *lon-8::gfp *in the lateral and ventral part of the tail hypodermis. These are, from 1–7: V6.pppaa, T.aa, R6.p, T.apapa, R7.p, R8.p and R9.p. "Set" indicates the male tail seam. (C)-(D) left lateral view, (E)-(F) mid-plane view.

*C. elegans *males possess a copulatory tail consisting of nine bilaterally symmetric pairs of sensory structures called rays and two sharp, hardened structures called spicules [25]. The ray pairs have distinct identities with respect to morphology and chemosensitivity. As the name of the pathway suggests, Sma/Mab pathway mutants show extensive defects in ray specification. [16-19,26]. In addition, all Sma/Mab pathway mutants have crumpled spicules. Male mutant *dbl-1, daf-4, sma-2*, *sma-3*, *sma-4 *and *sma-6 *animals exhibit dorsal to ventral transformations of rays 5, 7 and 9, which take on the fate of their anterior neighbors 4, 6, and 8 and often fuse with them [16-19,26]. *dbl-1 *overexpression has the opposite effect, dorsalizing ray 4 which can lead to fusions of rays 3 and 4 [17]. If *lon-8 *is acting in parallel to the Sma/Mab pathway in body size regulation, does it also affect male tail development? To address this question, we analyzed the morphology of the male rays using a *him-5(e1490) *mutant background. We found that *lon-8(hu187) *and *lon-8(hu188) *mutants have extensive morphological ray defects, but we did not observe any defects of the spicules. All animals examined have similar ray defects but the severity of the phenotype is variable: in some animals the rays appear grossly thickened but can still be identified. In these cases, the correct positional identity of the rays does not seem to be affected. In most males, the rays have such an abnormal appearance that they can no longer readily be distinguished (Figure 6). All rays appear morphologically abnormal, but rays 5 and 6 are most severely affected, whereas rays 8 and 9 are less deformed than their more anterior neighbors. Surprisingly, *lon-8(hu187) *and *lon-8(hu188) *males can mate, suggesting that ray sensory function is intact even though ray morphology is grossly abnormal. This phenotype is most reminiscent of the phenotype of mutants in a class of 8 genes that give a Ray Abnormal Morphology (Ram) phenotype [27]. These genes act after the cell lineage is complete to regulate the apposition of the cuticle around the rays. Although mutations in the *ram *genes result in an abnormal, lumpy ray morphology, mutant animals appear otherwise normal and can mate. Finally, we examined the expression of the *lon-8::gfp *transcriptional reporter described above in *him-5 *animals (Figure 6). In males, the reporter is expressed in hyp4 and hyp7 like in hermaphrodites. In addition, GFP is expressed in the nuclei V6.pppaa, T.aa, R6.p, T.apapa, R7.p, R8.p and R9.p. These cells fuse and make up the ventral part of the male tail hypodermis [25]. In conclusion, LON-8 is expressed in hypodermal tissues that surround the developing rays.

### Functional interaction between *lon-8 *and the Ram genes *dpy-11 *and *dpy-18*

**Figure 7:**
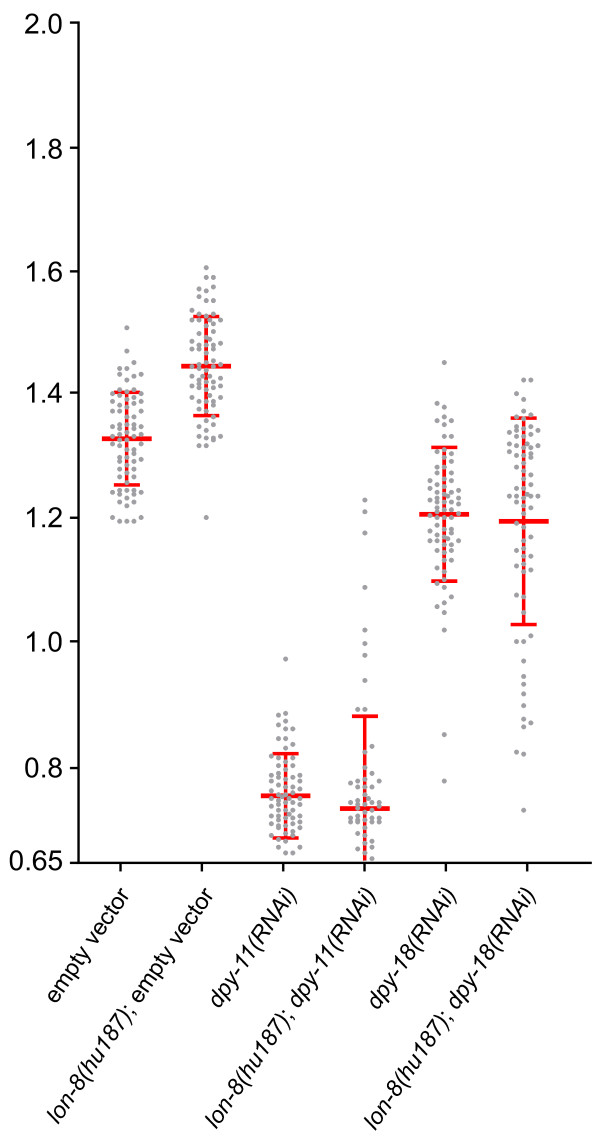
**Loss of *dpy-11 *or *dpy-18 *suppress the *lon-8 *phenotype**. Animals were grown on the indicated dsRNA expressing bacteria and were measured as described in Methods. At least 75 animals were measured for each strain. Each dot represents one animal. Average length and standard deviation are indicated. *dpy-11 *and *dpy-18 *RNAi in wild type and *lon-8(hu187) *backgrounds result in equal body lengths (p > 0.05).

In addition to their role in male tail development, some of the mutations that cause a Ram phenotype also have a significant effect on body size, causing a dumpy (Dpy) phenotype [27]. Two of the genes affected by these mutations, *dpy-11 *and *dpy-18*, encode enzymes required for the posttranslational modification of cuticle collagens [28-30]. As mutations in *dpy-11 *and *dpy-18 *cause a male tail phenotype similar to *lon-8*, we investigated whether they might interact with *lon-8 *in body size regulation. We found that both *dpy-11(RNAi) *and *dpy-18(RNAi) *completely suppress the *lon-8 *body size phenotype (Figure 7), strongly suggesting that the function of these three genes are intimately linked.

### LON-8 has been highly conserved throughout nematode evolution

**Figure 8:**
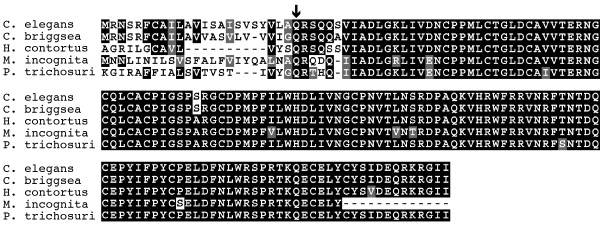
**Conservation of LON-8 in nematodes**. Alignment of the predicted LON-8 sequence from five Rhabditid nematode species. *C. elegans*, *C. briggsae *and the closely related vertebrate parasitic Strongyloid nematode *Haemonchus contortus *belong to the family of Rhabditine nematodes. *Parastrongyloides trichosuri *and *Meloidogyne incognita *are more distantly related Tylenchine nematodes. The site of cleavage of the predicted signal peptides is indicated.

Sequence similarity searches of the NCBI protein database and a recently established database of parasitic nematode expressed sequence tags (ESTs) [31], indicated that LON-8 is a nematode specific protein. Clear LON-8 homologs were found in *Caenorhabditis briggsae*, *Haemonchus contortus*, *Pristionchus pacificus*, *Parastrongyloides trichosuri*, *Meloidogyne incognita *and *Brugia malayi*, representing each of the three clades of the order Rhabditida [32,33]. When the sequences from the different nematode species are aligned, the only major divergence is in the N-terminal part of the protein (Figure 8). Despite this difference in amino acid sequence, all the putative N-termini are predicted signal peptides. Therefore, the function of this part of the LON-8 protein is conserved even though the sequence has diverged. This suggests that *lon-8 *has an important function in all Rhabditid nematodes.

## Discussion

### *lon-8 *regulates body size independently of the Sma/Mab pathway

In this study, we describe *lon-8*, a novel regulator of body size. So far, all the *lon *genes that have been cloned show clear interactions with the Sma/Mab pathway [11-14]. Our epistatic analysis of *lon-8 *and other body size mutants imply that *lon-8 *is an exception in that it is functioning in parallel to the Sma/Mab pathway. We observed that *dbl-1 *suppresses the *lon-8 *body size phenotype, indicating that *lon-8 *does not function downstream of *dbl-1*. Furthermore, the finding that *lon-8 *expression is not affected in *dbl-1 *or *sma-6 *mutants suggests that it is not a transcriptional target of the DBL-1 pathway. The suppression of the *lon-8 *phenotype by *dbl-1 *could also be explained by a function of *lon-8 *upstream of *dbl-1*. Although this cannot be excluded, the body size phenotypes of double mutant combinations of *lon-8 *and the three different *lon *genes that are part of the DBL-1 pathway argue against this possibility. We found that *lon-8 *enhances the body length of these *lon *mutants by a similar proportion, an additive effect which would not be expected if they were in a linear pathway. Taken together, the evidence suggests a parallel function of LON-8 and the Sma/Mab pathway in body size regulation.

### *lon-8 *regulates body size primarily during larval and early adult stages

The *lon-8 *body size phenotype is more subtle than those of the other *lon *genes; whereas *lon-1(e185) *animals are approximately 30% longer than wild-type, and *lon-3 *animals about 25%, *lon-8 *mutants have a more modest increase of 10% of the wild-type body length on average. Another distinction is that *lon-8 *appears to be affecting growth largely during larval development, whereas *lon-1 *acts throughout larval and post-larval growth (this study, [11,14]) and *lon-3 *acts primarily during late larval stages [11,12]. In addition, *lon-1 *partially exerts its effects by regulating hypodermal ploidy, which accounts for most, if not all of adult growth [2,11,14]. The increase in body size of *lon-8 *mutants shows no clear correlation with the nuclear content of the hypodermis. Taken together with the finding that the growth of *lon-8 *mutants is affected during larval development and early adulthood rather than throughout adult growth, we hypothesize that *lon-8 *is involved in regulating growth processes that are not dependent on hypodermal nuclear content.

### The *lon-8 *male tail phenotype is indicative of a function for LON-8 in cuticle retraction

*lon-8 *is the only *lon *gene studied so far to affect the development of the male tail. Although the rays are severely deformed in *lon-8 *animals, they do not seem to exhibit cell fate alterations as Sma/Mab pathway mutants do [17-19,26]. In Sma/Mab mutants, the identities of specific rays are altered to resemble that of their anterior or posterior neighbor, and this leads to fusions between rays with similar fates. In *lon-8 *mutants, the rays appear to be deformed rather than misspecified and that is why they can no longer readily be distinguished. The spicules do not look affected and the males can mate. This phenotype is quite different from that of Sma/Mab mutants, again suggesting that *lon-8 *is not acting in this pathway. Rather, the *lon-8 *phenotype resembles the *ram *mutant phenotype [27]. Animals with a Ram phenotype have a defect in the extension of the rays and this leads to abnormal male tail morphology. Although the mutant rays are lumpy, their sensory function seems to be unaffected as *ram *males can mate. We hypothesize that, like the *ram *genes, *lon-8 *is required for the retraction of the hypodermis that accompanies the terminal differentiation of the rays.

### *lon-8 *genetically interacts with enzymes required for the modification of cuticle components

A number of the *ram *genes have been found to encode components of the extracellular cuticle (reviewed in [34]). Two of the genes that give a Ram phenotype are the previously identified genes *dpy-11 *and *dpy-18*, which encode enzymes synthesized by the hypodermis required for the posttranslational modification of cuticle components [28-30]. Epistatic analysis shows that loss-of-function of these genes can fully suppress the *lon-8 *body size phenotype, indicating that *dpy-11 *and *dpy-18 *function genetically downstream of *lon-8*. There are many possible hypotheses as to the actual mechanism of the interaction. Taking into account that LON-8 is most likely a secreted component of the cuticle, it might for instance function to modulate the conformation or localization of targets of DPY-11 and DPY-18.

### The conservation of LON-8 implies that it performs an important function in nematodes

So far, the *C. elegans *and *C. briggsae *genomes have been fully sequenced and made publicly available. In addition, over half a million ESTs have been generated from around 40 species of nematodes representing all but one clade [35], enabling extensive cross-species comparisons. Although the Rhabditid nematode species share morphological similarity, there is a great deal of genetic diversity between them. For example, *C. elegans *and *C. briggsae*, which are the most closely related of the set and are morphologically indistinguishable, are separated by an estimated 100 million years of evolution, and 10% of their genes differ [36]. Analysis of nematode ESTs showed that LON-8 is highly conserved within species of the order Rhabditida, which is one of the major nematode orders and contains free living as well as animal and plant parasitic nematodes [32]. LON-8 mediated regulation of body size may thus be an essential feature of nematode development.

## Conclusion

We have identified a novel gene that controls body size and male ray morphology in *C. elegans *and is conserved in nematodes. Although *dbl-1 *signaling regulates both body size determination and male tail development, *lon-8 *does not appear to be an effector of the Sma/Mab pathway. The presence of a secretion signal and the fact that a *lon-8 *promoter fusion is expressed in the hypodermis suggest that LON-8 is a secreted component of the extracellular cuticle. The interaction between *lon-8 *and genes encoding cuticle collagen modifying enzymes further suggests that LON-8 may be important for cuticle structure, thereby influencing body size as well as male ray extension.

## Methods

### General methods and strains

Strains were cultured using standard methods [10] and maintained at 20°C. The Bristol N2 strain was used as a wild-type standard strain in all experiments. All crosses were carried out according to standard methods [37]. The mutant alleles used are listed below by linkage group (LG) and have been described by [10] unless otherwise stated: (LGII): *sma-6(1482)*, (LGIII): *lon-1(e185)*, (LGV): *lon-8(hu187)*, *lon-8(hu188)*, (this study), *lon-3(e2175), dbl-1(nk3) *[16], *him-5(e1490)*, (LGX): *lon-2(e678)*. The following transgenic lines were used in this study: BW1940 *ctIs40 [dbl-1xs] *[17], JR667 *wIs51 *[22], KN746 *huEx96 *(this study), KN905 *huEx129 *(this study).

### Sequencing of *lon-8*

To amplify the *lon-8 *cDNA from a yeast-two-hybrid cDNA library (a gift of M. Walhout and M. Vidal) we used a primer directed to the 5' side of the pPC86 poly-linker with the *lon-8 *primer CTGCAGAATTACTGACTGTTGGGATC and a primer directed to the 3' side of pPC86 with the *lon-8 *primer CAGTCAGTAATTGCG-GATTTG. All amplified products were purified and sequenced from both directions to obtain the full-length *lon-8 *cDNA sequence.

To sequence the *lon-8 *transcript present in wild type and *hu187 *animals, we used 1 μg of total RNA from N2 and *hu187 *(see below) to generate cDNA by RT-PCR using an oligo-dT primer. We then amplified the *lon-8 *cDNA using primers for the SL1 sequence and CTGCAGATTCCTCGCTTTCGCTG corresponding to the 3' end of the fourth exon of *lon-8 *and sequenced the purified products from both directions to obtain the wild-type and *hu187 *cDNA sequences.

### Isolation and characterization of *lon-8 *alleles

The deletion alleles *lon-8(hu187) *and *lon-8(hu188) *were generated by constructing and screening a deletion library as described by [38]. The primers used to identify and sequence the *hu187 *deletion allele were TGCAATAGGTGTACGAAAAG and CCACAACACATACATTCCAT as the external pair and GAGTGGAATGCTAATAGGGA and GTGAAGCAGCGTATCAACTA as the internal pair. The primers used to identify and sequence the *hu188 *deletion allele were TTTTCTTTGAGCCGCAAAAT and CAGGTAACGCCATGAGGAAT as the external pair and CAAAGGAGGTCGAAAGTGGA and CCATTCTGGCAGTGATCTCC as the internal pair.

### Body size measurements

Strains were synchronized by gently flushing 9 cm plates of a mixed stage population to remove all adults and larvae, leaving the unhatched eggs. One hour later the plates were gently flushed to collect the larvae hatched within this interval. For the growth curve, synchronized animals were kept at 20°C and one plate of each strain was used for measurements at each time point. A minimum of 75 random animals was sampled every time. For the epistatic analysis an N2 control was taken along with each synchronization to ensure consistent results as body size may vary due to external conditions such as humidity [11]. To measure, the animals were mounted on 2% agarose slides containing 10 mM Sodium azide and immediately photographed at a magnification of 33.5 times using a Zeiss Axioplan2 microscope and attached Axiovision digital camera. Body length was determined by using Object Image 2.13 software. The data was analyzed using GraphPad Prism.

### *lon-8p::gfp *fusion constructs

To generate the *Plon-8::gfp *fusion construct pGS25, sequence corresponding to the 2 kb upstream region up to the second exon of the predicted *lon-8 *transcript was amplified from N2 genomic DNA using primers (*Pst*I)-CTTGACCGTAGAGCTTCC and (*Pst*I)-CAAATCCGCAATTACTGACTG. This fragment was cloned into the pPD95.69 GFP insertion vector (A. Fire, personal communication) using the *Pst*I cloning site. To generate the construct excluding the predicted signal peptide, pGS26, two fragments were amplified from pGS25: the cloned *lon-8 *upstream sequence up to the predicted ATG using the reverse primer (*Bgl*II)-ATTCCTCATGGCGTTAC, and a fragment consisting of the end of the first exon up to the beginning of the second exon using the forward primer (*Bgl*II)-AGTGAGTTGTTTAC. The digested fragments were ligated simultaneously into pPD95.69 using the *Pst*I cloning site.

Both fusion constructs were injected at a concentration of 50 ng/μl into *dpy-20(e1282) *and N2 animals using 50 ng/μl pMH86 (containing wild type *dpy-20*) or 20 ng/μl of pPY20 (P.T. Yang, personal communication) as co-injection markers. pPY20 (*Pmyo-2::tomato*) expresses the red fluorescent tomato protein [39] in the pharynx. Several lines were generated that showed similar expression patterns. *huEx96 *and *huEx129 *were selected for further analysis.

### Northern blots

Total RNA was collected from mixed stage populations of N2, *lon-8(hu187)*, *lon-8(hu188)*, *lon-1(e185)*, *ctIs40 [dbl-1xs]*, *dbl-1(nk3) *and *sma-6(e1482)*. Animals were lysed for 5 minutes at 60°C in buffer containing 2 mg/ml proteinase K. RNA was isolated using Trizol LS (Invitrogen) followed by chloroform extraction and isopropanol precipitation. 10 μg of total RNA was loaded into each lane of a 6% formaldehyde gel. After electrophoresis, RNA was transferred onto Bio-Rad Zeta-Probe membrane. The *lon-8 *probe was generated by amplifying the *lon-8 *transcript from a cDNA library using the primers CCCGGGCATGAGGAATTCGCGGTT and CCCGGGTATGATATGGAATTATCG. The *lon-1 *probe was generated by amplifying a 250 bp fragment of exon 6 from N2 genomic DNA using the primers GATGCCAGCTTTCACCTG and CTCTCATTCGGAACCCGTC. The *eft-2 *probe was generated by amplifying a 400 bp fragment of exon 4 from N2 genomic DNA using the primers GCACAATCGTCTTCACTGTACC and GTAAGAACCGAAGCGTAGAAC.

### Endoreduplication measurement

We synchronized populations of N2, *lon-1(e185)*, and *lon-8(hu187) *as described above. After 120 hours, the animals were collected and fixed in Carnoy's solution overnight. The animals were then either stained with DAPI and analyzed as described [2], or stained with PI and immediately analyzed by confocal microscopy as described [23]. For each animal, we made one confocal stack of the tail area between the V5.ppppp and V6.ppppp seam cell nuclei. The DNA content of all the hypodermal nuclei located between these seam cells (8 nuclei per animal) was measured as defined by the sum of PI intensity. We then divided this number by the average of the PI intensity of at least four neuronal nuclei of the ventral nerve cord from the same confocal stack to obtain an average ratio of hypodermal to neuronal DNA content for each animal.

### RNAi experiments

Bacterial clones expressing dsRNA of selected genes were picked from the Ahringer lab RNAi library [40]. Fifty wild type or *lon-8(hu187) *L4 larvae were placed on plates containing empty vector, *lon-8, dpy-11 *or *dpy-18 *dsRNA expressing bacteria, grown overnight at 15°C and transferred to fresh RNAi plates. Populations were synchronized by removing the animals after 7 hours, leaving the unhatched progeny. These animals were grown at 15°C for 190 hours, which is the equivalent of 120 hours of growth at 20°C [41], then collected and measured as described above.

## Authors' contributions

G.S. carried out all experimental procedures, H.C.K. participated in the design of the study and supervised the work. M.C.B. was instrumental in discovering Y59A8B.20. G.S. and H.C.K. prepared the manuscript. All authors read and approved the final manuscript.
